# Developing a new set of temperature calibration materials for high-temperature thermal analyses

**DOI:** 10.1007/s10973-025-15191-8

**Published:** 2026-01-04

**Authors:** Kil-Won Moon, Jiwon Park, Joo-Hee Kang, Chang-Seok Oh, Jack Prothero, Ursula Kattner, Carelyn Campbell

**Affiliations:** 1 Materials Science and Engineering Division, National Institute of Standards and Technology, 100 Bureau Drive, Gaithersburg, MD 20899 USA; 2 Korea Institute of Materials Science, 797 Changwondaero, Changwon, Gyeongnam 51508 Republic of Korea; 3 Statistical Engineering Division, National Institute of Standards and Technology, 100 Bureau Drive, Gaithersburg, MD 20899 USA

**Keywords:** Thermal analysis (DTA/DSC), High-temperature calibration alloys, Onset temperature of melting (T_onset_)

## Abstract

A new set of Research Grade Test Materials (RGTMs) has been developed for accurate temperature calibration in high-temperature differential scanning calorimetry (DSC) and differential thermal analysis (DTA). The candidate eutectic binary alloys and their melting temperatures (m.p.) are Ni-41Nb (The alloy compositions are presented as mole fraction percentages, and the purity of elements and alloys is represented in mass fraction percentages) (1181 °C), Co-13Nb (1240 °C), Fe-10Nb (1372 °C), Rh-23.5Si (1421 °C), and Ru-28Si (1535 °C). This set of calibration alloys will cover the temperature range between the melting points of gold (1064 °C) and nickel (1455 °C) and help eliminate uncertainties caused by the oxidation of Ni and its eutectic reaction of L (Ni) + NiO. Additionally, DSC/DTA instruments that require intermediate temperature calibration between gold and nickel can now accurately perform temperature calibration using these new RGTMs. After calibrating with RGTMs instead of Ni, the estimated variance has improved from 9.16 to 0.65 °C.

## Introduction

High-temperature materials (defined here as having melting temperatures between 1100 to 1500 °C), such as superalloys and high-entropy alloys, are being developed to enhance fuel efficiency and reduce pollution in gas turbines, rocket engines, and jet propulsion applications [1]. These materials also play a vital role in advancing manufacturing processes that require durability against high-temperature scaling and mechanical stress. The design of high-temperature materials depends on their thermodynamic, kinetic, and mechanical properties. To maximize efficiency, establishing accurate optimal operating conditions based on temperature is equally important as developing the new alloy. Therefore, a new set of high-temperature calibration alloys is needed for precise measurements of phase transition temperatures.

Typical instruments to measure phase transformation temperatures are differential scanning calorimeter (DSC) and differential thermal analyzer (DTA). The calibration of temperature measurements depends on using the established melting points (m.p.) of elemental standards (refer to Group A in Table 1) [2–4]. However, the availability of suitable high-temperature reference materials is limited. For instance, there is a substantial difference between the melting points of Au (m.p. 1064 °C) and Ni (m.p. 1455 °C). Furthermore, the oxidation of the Ni reference material introduces a significant level of uncertainty due to the continuous decrease in apparent Ni melting temperature until the L ↔  (Ni) + NiO eutectic reaction is reached. This can lead to significant errors if Ni is the last data point with a considerable interval. Additionally, if the maximum temperature of the instruments is between 1100 and 1500 °C, an appropriate elemental standard is not available for accurate measurements using interpolated calibration.

**Table 1:** Summary of the linear fit with the 0.99 confidence boundaries depending on the heating rates for elemental standards (A), validation alloys (B), and RGTM alloys (C). The intercepts represent the equilibrium melting temperatures

Group	Substance/mole fraction %	Recommended m.p./°C	Intercept temperature/°C	Lower bound of intercept (confi. level: 0.99)/°C	Upper bound of intercept (confi. level: 0.99)/°C	Slope/min	Lower bound of slope (confi. level: 0.99)/min	Upper bound of slope (confi. level: 0.99)/min
**A**	Sn	**232**	**2.297E + 02**	2.292E + 02	2.301E + 02	**3.460E-02**	− 7.000E-03	7.700E-02
	Bi	**271**	**2.686E + 02**	2.685E + 02	2.688E + 02	** − 4.400E-02**	− 5.400E-02	− 3.300E-02
	Al	**660**	**6.552E + 02**	6.544E + 02	6.560E + 02	**6.950E-02**	− 3.000E-03	1.420E-01
	Ag	**962**	**9.567E + 02**	9.564E + 02	9.571E + 02	**2.740E-02**	− 4.000E-03	5.900E-02
	Au	**1064**	**1.060E + 03**	1.060E + 03	1.061E + 03	**1.700E-02**	− 2.800E-02	6.300E-02
	Ni	**1455**	**1.452E + 03**	1.451E + 03	1.452E + 03	**5.390E-02**	1.610E-02	9.160E-02
	Co	**1495**	**1.491E + 03**	1.490E + 03	1.492E + 03	**1.270E-02**	− 5.900E-02	8.400E-02
**B**	Al-28Ge	**424**	**4.208E + 02**	4.202E + 02	4.214E + 02	** − 2.290E-02**	− 7.000E-02	2.400E-02
	Ag-40Cu	**779**	**7.745E + 02**	7.736E + 02	7.754E + 02	**4.800E-02**	− 3.900E-02	1.340E-01
	Ni-45Pd	**1237**	**1.233E + 03**	1.233E + 03	1.234E + 03	** − 5.100E-02**	− 1.290E-01	2.700E-02
**C**	Ni-41Nb	1181	1.177E + 03	1.176E + 03	1.177E + 03	− 1.460E-01	− 1.991E-01	− 8.869E-02
	Co-13Nb	1240	1.236E + 03	1.236E + 03	1.237E + 03	− 1.578E-01	− 2.096E-01	− 1.073E-01
	Fe-10Nb	**1372**	**1.368E + 03**	1.368E + 03	1.368E + 03	** − 7.886E-02**	− 9.975E-02	− 5.784E-02
	Rh-23.5Si	**1421**	**1.417E + 03**	1.416E + 03	1.418E + 03	** − 1.460E-01**	− 2.210E-01	− 6.934E-02
	Ru-28Si	**1535**	**1.531E + 03**	1.530E + 03	1.532E + 03	** − 2.935E-02**	− 1.337E-01	6.103E-02

An external oxygen-getter system or an internal oxygen trap insert could be installed to reduce the effects of oxidation on Ni standards. However, the test conditions may still be uncertain unless the oxygen levels are monitored, which is not a standard practice for these measurements. The best solution is to use suitable calibration materials for high-temperature calibration. This study aims to identify appropriate metal alloys as Research-Grade Test Materials (RGTM) and suggest calibration methods to enable accurate high-temperature measurements in the range from 1100 to 1500 °C. It should be noted that Pd (m.p. 1555 °C) was not included in the present study because its melting temperature is outside the temperature range of interest.

In general, alloys have less distinct melting and cooling signals compared to pure elements. Additionally, these signals are strongly dependent on the microstructure and sample history. It is crucial for RGTMs to have reliable and unique DSC/DTA curves. Therefore, the alloy needs to be free of phase transformations in the solid state, and melting should occur rapidly, independent of composition variation. To meet these criteria, alloys with eutectic invariant reactions with a fast reaction between liquid and solids and with a melting temperature that is insensitive to minor composition variations were considered [5].

For DSC/DTA temperature calibration, only the melting curves will be considered in this paper to avoid complications from nucleation issues during solidification. Multiple melting experiments will be performed to obtain statistically reliable data. In addition, the first melting curve will also be discarded to avoid complications from the sample history and contact problems between the sample and the crucible [5, 6]. The equilibrium melting temperatures of RGTMs were also determined by fitting the observed melting temperature versus the heating rates and extrapolation to zero heating rate [6, 7].

## A new method to determine the melting temperature

### *Definitions of melting points: T*_*onset*_* or T*_*extrap*_

**Fig. 1:**
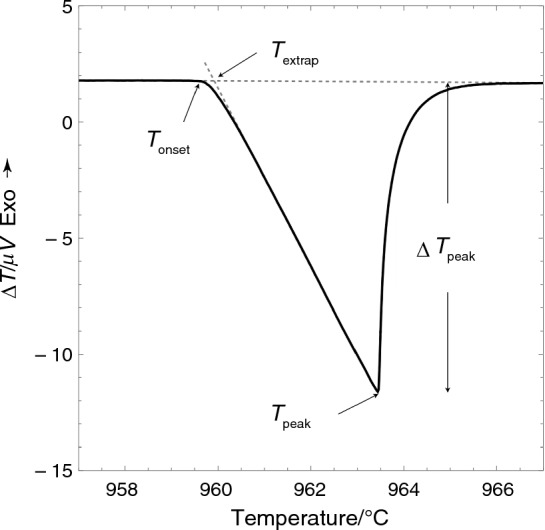
A silver melting curve typically illustrates three temperature definitions: onset, extrapolation, and peak temperature. ΔT represents the difference between the sample temperature and the reference temperature

The ASTM E967-08R14 standard [8] defines the melting temperature as T_extrap_, an extrapolated onset temperature determined from the baseline and peak slope, as shown in Fig. 1. On the other hand, the ASTM E794-01 standard [9] and NIST Best Practice Guide SP 960–15 [5] define the melting point as T_onset,_ the first detection of the melting sign in Fig. 1. There is a potential for a temperature discrepancy between the true and determined melting temperatures since the extrapolated onset temperature depends on the peak slope, which depends on the sample mass, thermal lag, and the melting properties of the material, and is not linked to a specific thermodynamic property. Moon and coworkers [10] demonstrated that the ∆T_peak_, as defined in Fig. 1, increases with increasing heating rate, and the peak slope only correlates with instrument sensitivity; it is not associated with a specific thermodynamic property. Therefore, this paper defines T_onset_ as the melting point closest to the true melting point.

### *New determination method of T*_*onset*_*: assessing residuals in the forward step line equations*

It is essential to have a straightforward, adaptable, and reliable method for determining T_onset_ to avoid errors resulting from human subjectivity and extrapolation methods. A new method has been developed because when melting begins in pure elements or eutectic alloys, the melting signal is an endothermic event in ∆T and continues until the melting is complete. We have focused on these facts and developed a strategy within the interpolating range using a downward endothermic peak.

**Fig. 2:**
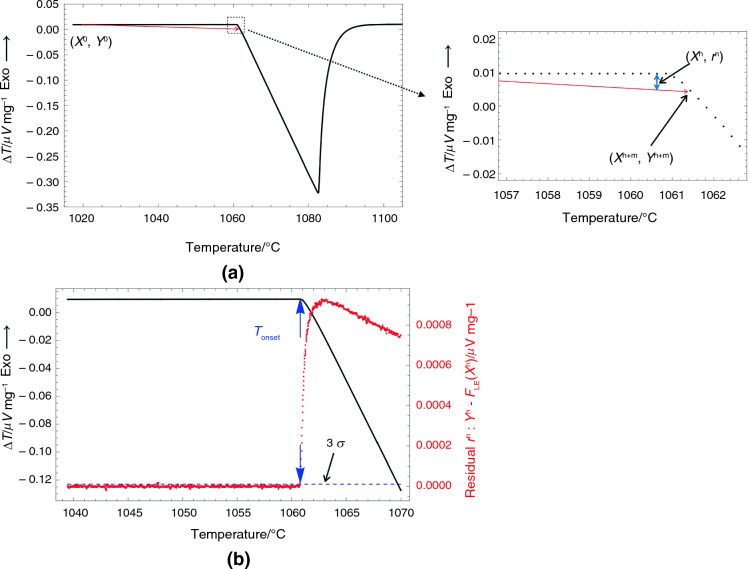
**a** shows a typical T vs. ∆T DSC curve of Au tested under Ar flow with a heating rate of 2 °C min^−1^. **b** shows the region of interest in (a) with the T versus ∆T curve and T versus r^n^. The residual r^n^ is the difference of y^n^ and the F_LE_(x^n^) line between (x_0_,y_0_) and (x^n+m^, y^n+m^)

First, the region of interest was selected near the melting area, and the lowest melting temperature was estimated from the measurements. For example, in Fig. 2a, the selected region of interest is around 1018 to 1105 °C, with the estimated lowest melting temperature being about 1060 °C. Then, the basal point (x^0^, y^0^) was calculated based on the mean value or the bi-weighted location of the first 5% of data from the region of interest. The equation for the line F_LE_(x^n+m^) was calculated between (x^0^, y^0^) and (x^n+m^, y^n+m^), where ‘m’ represents the data points following x^n^. The default value of 'm' has been empirically established as 4.

In the next step, the residual (r^n^) was evaluated by finding the difference between (x^n^, F_LE_(x^n^)) and (x^n^, y^n^). The data point (x^1^, y^1^) represents the temperature (x) and ∆T (y) of the next 5% of data from the region of interest. The calculated residuals (x^n^, r^n^) are plotted as the red curve in Fig. 2b. Finally, the value T_onset_ was determined from the red curve where the residual was more than three times the standard deviation (σ). The standard deviation was calculated from x^1^ to the estimated lowest melting point.

Sometimes, it can be difficult to determine T_onset_ from noisy data due to multiple T_onset_ values or an overestimated T_onset_ caused by a large 3σ. In these situations, T_onset_ was chosen from the last significant value of 3σ and the reduced confidence range, for example, 2σ. Additionally, when creating the plot of the calculated residuals (x^n^, r^n^), using a 'm' number larger than four reduced the noise level, for example, empirically selecting a number between seven and nine.

### Determination of the equilibrium melting temperature

In DSC/DTA measurements, the quantity of heat flow in a sample depends on several factors, including the geometrical arrangement of the sample and reference, the atmosphere, and the temperature range of operation. Heat flow can occur through conduction, convection, and radiation processes. The individual contributions of each process can impact the temperature differences and thermal lags during measurements between the sample, reference, and thermocouples. To ensure accurate calibrations, it is important to consider various details affecting the specimen's melting and the thermal lags inherent in the instrument. As the heating rate approaches zero, the thermal lags will be eliminated [5, 11].

**Fig. 3:**
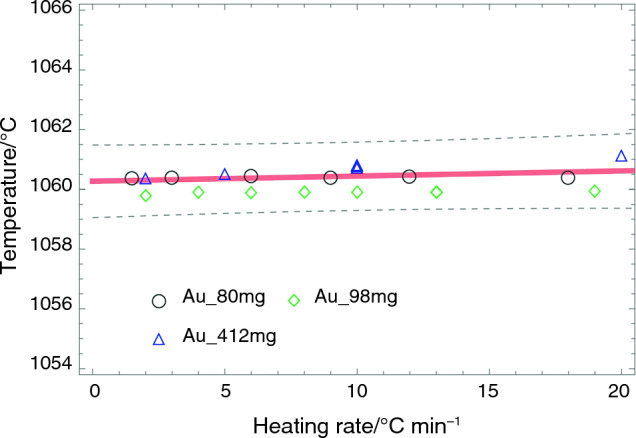
The figure illustrates a typical example of Au for determining the equilibrium melting temperature. The solid red line indicates the linear fit, and the dashed black lines represent the upper and lower of 0.99 confidence boundaries

For an RGTM alloy, it is paramount to determine the melting temperature in an equilibrium state. To get the equilibrium temperature, however, a slow heating rate or an isothermal experiment is required. Instead, a method that determines the melting point as a function of heating rates can be used to avoid these expensive experiments [6, 7]. In this function, the y-intercept, i.e., the function value at zero heating rate, will be considered the equilibrium melting point. Using the established linear function, this method makes temperature calibration feasible for all heating rates. Figure 3 shows typical results of Au to determine the equilibrium melting temperature. The measured melting temperature is 1060.3 ± 0.5 °C, the compensated melting temperature based on the calibrated linear fit is 1064.2 °C, and the recommended melting temperature is 1064 °C.

## Experiments

The experimental procedure was conducted in three stages. First, an elemental screening was performed to select candidate alloys and evaluate them as potential RGTMs. Second, a typical calibration procedure using elemental standards was performed, and the results were validated by testing Al-28Ge, Ag-40Cu, and Ni-45Pd alloys. Finally, RGTM's melting temperatures were determined based on the equilibrium melting temperature and validated using additional off-eutectic alloys.

All alloy ingots were prepared from pure elements in an arc melter, with the purity of elements being better than a mass fraction of 99.9%. The purity of the cast alloys was determined by inductively coupled plasma atomic emission spectroscopy (ICP-AES). Netzsch^1^^*^ DSC (model 404F1, flow-compensated DSC) with a DSC head was employed to evaluate the melting temperature. The sample mass was typically about 0.1 g, and the gas flow rate was 0.04 L min^−1^ of 99.999% Ar.

When evaluating the candidate alloys, the samples were heated at 5 or 10 °C min^−1^ and cooled at 60 °C min^−1^ to promote finer eutectic structures. However, to determine the equilibrium melting temperatures, the heating rates were varied between 0.5 and 20 °C min^−1^ while maintaining a constant cooling rate of 60 °C min^−1^. Note that the cooling rates were appropriately regulated within the specified cycling temperature ranges. Standard metallographic examination was performed on RGTMs and off-eutectic alloys. Phase compositions were identified using energy-dispersive X-ray analysis in the scanning electron microscope (SEM).

## Results

### Results of element screening and evaluation of pilot alloys

A screening was conducted to identify the most suitable metallic elements for RGTMs. Out of the 118 elements, 96 were excluded because they were unstable, radioactive, toxic, had low boiling points (below 2000 °C), had high vapor pressure at their melting points (more than 10 Pa), or were highly oxidized (with oxide formation energy greater than − 500 kJ mol^−1^). The remaining 22 elements were used to investigate candidate alloys with eutectic reactions between 1100 and 1600 °C.

Using the ASM alloy phase diagram database [12], 54 eutectic reactions were identified. To determine the most suitable alloys, they were grouped within 100 °C intervals. Ten pilot alloys were selected based on their melting point range and compositional stability of the eutectic reaction range.

### Assessments of pilot alloys based on 100 °C intervals

Ni-21Si (m.p. 1156 °C) and Ni-41Nb (m.p. 1179 °C) alloys were examined in the range from 1100 to 1200 °C. Both alloys exhibited a solid–liquid phase transition with a stable baseline before melting. However, at a heating rate exceeding 10 °C min^−1^, the polymorphic reaction of Ni-21Si at 1127 °C led to insufficient baseline stability. For both alloys, the wetting angles measured were greater than 90°, and the changes in sample masses were less than 0.1 mg, which is less than 0.1% of the sample mass.

Between 1200 and 1300 °C, Co-13Nb (m.p. 1241 °C) and Ni-15Nb (m.p. 1299 °C) were tested. Both alloys exhibited a wetting angle greater than 90°, and no interaction was observed between the samples and crucibles. Some oxidation and vaporization were noted, with changes in sample mass less than 0.1 mg, which is less than 0.1% of the sample mass. However, excessive amounts of black oxidized powders of Ni-15Nb were found in the crucible.

For temperatures ranging from 1300 to 1400 °C, the materials Nb-37Co (m.p. 1363 °C) and Fe-10Nb (m.p. 1371 °C) were examined. The wetting angle of Nb-37Co and Fe-10Nb was greater than 90°, and vaporization or interaction between the sample and the crucible was not observed, but black oxide films covered both sample surfaces. The melting temperature of Nb-37Co strongly depends on the cooling rates and was affected by an undulated baseline before melting, which was assumed to be caused by growing (Nb) needles in the NbCo µ-phase matrix.

In the 1400–1500 °C temperature range, we compared the characteristics of two eutectic alloys, Rh-23Si (melting point 1421 °C) and Fe-61Nb (melting point 1489 °C). Both alloys exhibited a well-defined solid–liquid phase transition with a stable baseline before melting. Rh-23Si showed minimal oxidation and vaporization. Additionally, the wetting angle was greater than 90°, and there was no interaction between the sample and crucible, nor any change in sample mass observed. On the other hand, the wetting angle of Fe-61Nb was less than 90°, and there was an interaction between the sample and the crucible. The Fe-61Nb sample experienced a mass gain of 0.8 mg, which is about 0.4% of the sample mass, indicating oxidation.

In the temperature range of 1500–1600 °C, the alloys Nb-45Rh (m.p. 1502 °C) and Ru-31Si (m.p. 1515 °C) were chosen. The recorded melting temperature of Nb-45Rh was 1478 °C, despite the reported eutectic temperature being 1502 °C. Both alloys exhibited a distinct solid–liquid phase transition. However, the baseline of Nb-45Rh was unstable (undulated) before melting, with a wetting angle of less than 90° and interaction between the sample and the crucible. On the other hand, Ru-31Si showed a wetting angle larger than 90°, with no interaction between the sample and the crucible. Nevertheless, the baseline of Ru-31Si was sometimes undulated before melting.

Based on the test results, five alloys that showed severe oxidation, vaporization, and a non-uniform baseline before melting were excluded from the candidate pool. The final selection of alloys is listed under Group C in Table 1. The ICP-AES results for the purity in mass fraction of the cast alloys of this final selection are as follows: Ni-Nb 99.9%, Co-Nb 99.6%, Fe-Nb 99.9%, Rh-Si 99.8%, and Ru-Si 99.7%.

### Preparing a precision temperature calibration

**Fig. 4:**
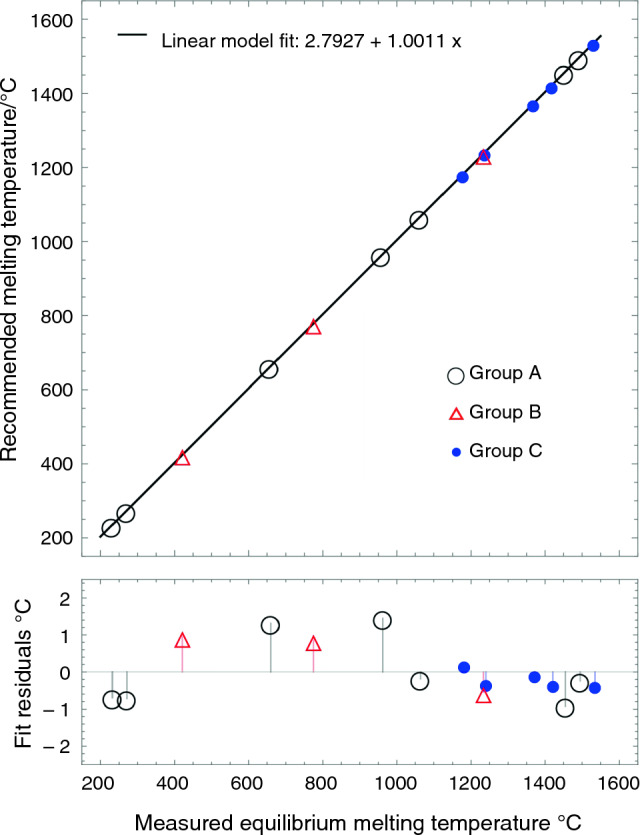
The figure shows the linear fit based on the elemental standards (black open circles), plotted together with the validation alloys (red open triangles), and the RGRMs (blue closed circles). The residual plots are shown as variances based on the linear model fit

A precise temperature calibration was conducted to determine the melting points of RGTMs with the extra oxygen trap insertion. In Fig. 4, the equilibrium melting temperature for the elemental standards is represented by black open circles, which is the result of the y-value at zero heating rate of a linear fit between various heating rates and T_onset_ (see Group A in Table 1 for the detailed linear fit results of each datum). The linear fit results of the equilibrium and recommended melting temperatures are as follows: the y-intercept is 2.793 °C, and the slope is 1.001 min, with standard errors of 0.847 °C and 0.001 min, respectively. The R-squared value and the estimated variance are 1.000 and 1.151 °C, respectively. Figure 4 also shows the fit residuals of elements. The fit residual of Ni is smaller than the ones of Al and Ag, indicating that the error of Ni could be acceptable. Note again that an oxygen trap insertion was used to minimize the oxidation effects of Ni. The estimated variances with and without employing oxygen trap insertion are 1.151 and 9.163 °C, respectively. Furthermore, the melting point of Co was also added with the oxygen trap insertion to improve statistical accuracy at the high-temperature endpoint.

The accuracy of the linear fit for elemental standards was validated by assessing the equilibrium melting temperature of three alloys: Al-28Ge, Ag-40Cu, and Ni-45Pd. These alloys are denoted by red open triangles in Fig. 4, and the linear fit results based on the equilibrium melting temperatures are listed under Group B in Table 1. The most significant fit residual among the validation alloys is 0.952 °C in the Al-28Ge (m.p. 425 °C) eutectic alloy. Note that this fit residual still falls within the estimated variance of the linear fit. As a result, the fitted model is deemed reliable and used to determine the equilibrium melting temperature of RGTMs.

### Determining the equilibrium melting temperatures of RGTMs

The recommended melting temperatures of five RGTMs have been determined based on the precise calibration, as shown in Fig. 4. In the figure, the equilibrium melting temperatures of RGTMs are represented by blue-filled circles. Group C in Table 1 summarizes the detailed linear fit with a 0.99 confidence interval. Note that the oxygen trap insertion has yet to be employed when measuring the melting temperatures of RGTMs. The five RGTMs and their assessed melting temperatures are Ni-41Nb (m.p. 1181 °C), Co-12.9Nb (m.p. 1240 °C), Fe-10Nb (m.p. 1372 °C), Rh-23.5Si (m.p. 1421 °C), and Ru-28Si (m.p. 1535 °C). The linear fit of the RGTMs and elemental standards, excluding Ni and Co, have an intercept of 2.748 °C and a slope of 1.001 min, with standard errors of 0.629 °C and 0.001 min, respectively. The R-squared value and the estimated variance are 1.000 and 0.651 °C, respectively. These melting temperatures were also validated using the off-eutectic alloys, with the results to be presented in the next section.

### Validating the equilibrium melting temperatures of RGTMs

**Fig. 5:**
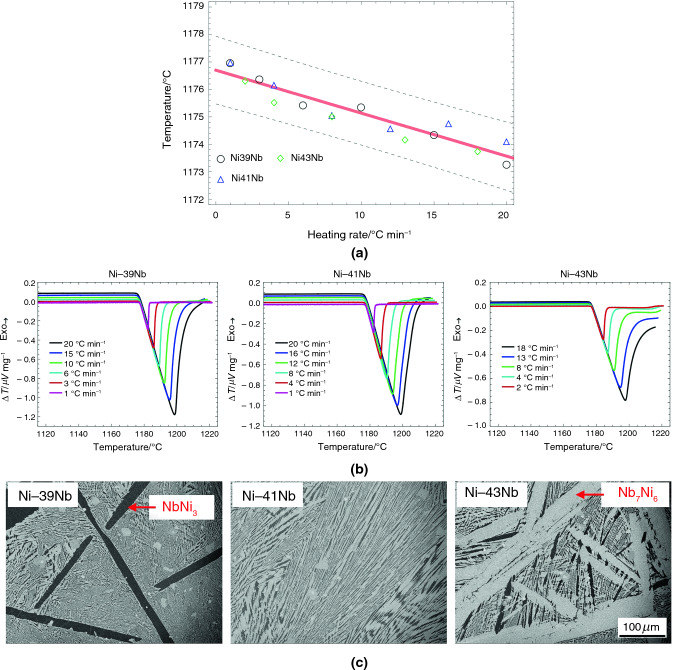
**A** Assessments of the equilibrium eutectic temperatures using Ni-39Nb, Ni-41Nb, and Ni-43Nb alloys, **B** the DSC results of Ni-39Nb, Ni-41Nb, and Ni-43Nb alloys depending on the heating rates, and **C** typical microstructures of Ni-39Nb, Ni-41Nb, and Ni-43Nb alloys

**Fig. 6:**
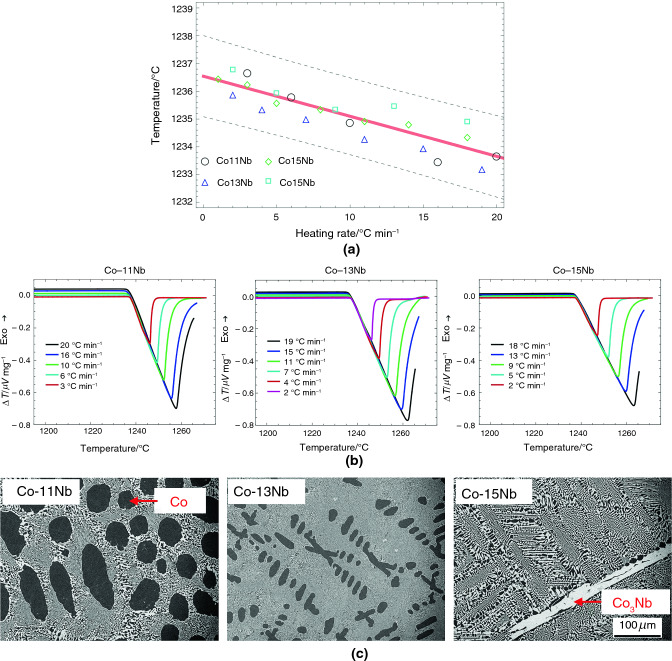
Comparison of linear fit results to calibrate DSC temperature using the test specimen set A (Sn, Bi, Al, Ag, Au, and Ni) and the set B (Sn, Bi, Ag, Au, Ni-41Nb, Co-13Nb, Fe-10Nb, Rh-23Si, and Ru-28Si)

**Fig. 7:**
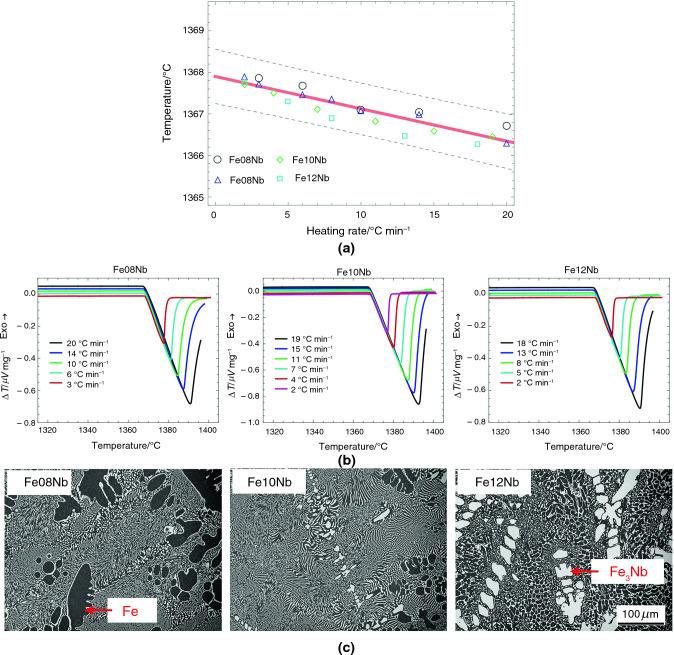
**A** Assessments of the equilibrium eutectic temperatures using Co-11Nb, Co-13Nb, and Co-15Nb alloys, **B** the DSC results of Co-11Nb, Co-13Nb, and Co-15Nb alloys depending on the heating rates, and **C** typical microstructures of Co-11Nb, Co-13Nb, and Co-15Nb alloys

**Fig. 8:**
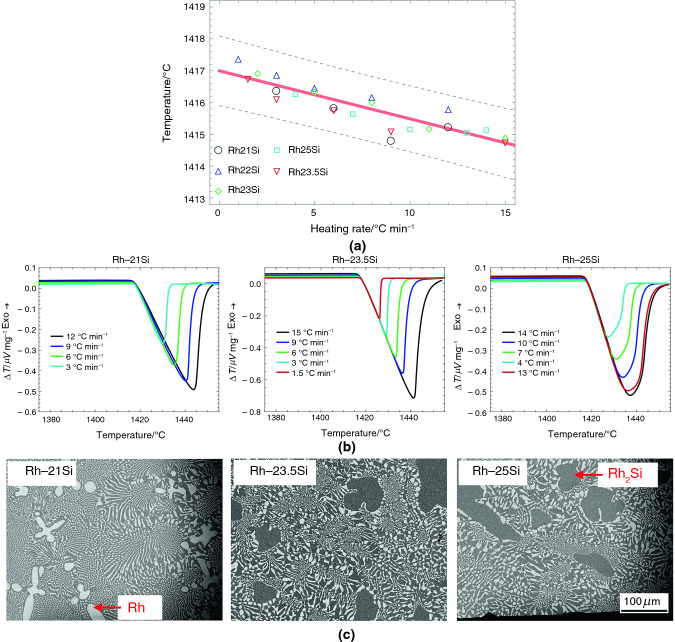
**A** Assessments of the equilibrium eutectic temperatures using Fe-8Nb, Fe-10Nb, and Fe-12Nb alloys; **B** the DSC results of Fe-8Nb, Fe-10Nb, and Fe-12Nb alloys depending on the heating rates; and **C** typical microstructures of Fe-8Nb, Fe-10Nb, and Fe-12Nb alloys

**Fig. 9:**
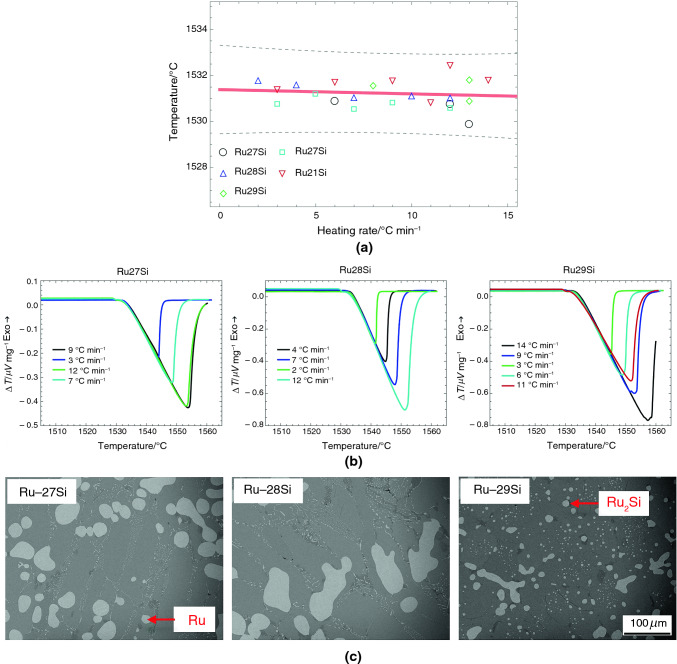
**A** Assessments of the equilibrium eutectic temperatures using Rh-21Si, Rh-22Si, Rh-23Si, Rh-23.5Si, and Rh-25Si alloys; **B** the DSC results of Rh-21Si, Rh-23.5Si, and Rh-25Si alloys depending on the heating rates; and **C** typical microstructures of Rh-21Si, Rh-23.5Si, and Rh-25Si alloys

Validating the melting temperatures of RGTMs is crucial, and employing another precise method will be the best approach. However, methods for determining melting temperatures above 1000 °C are limited. One way to validate the melting temperatures is by comparing them with the temperatures of the same invariant reaction of off-eutectic compositions since the RGTMs are based on eutectic alloys. Therefore, the equilibrium melting temperatures of five RGTMs and two extra off-eutectic alloys were assessed using the equilibrium T_onset_ of RGTMs. Figures 5–9 show a linear fit of equilibrium eutectic T_onset_ with the 0.99 confidence intervals (A), the DSC heating curves of RGTMs depending on the heating rates (B), and the related alloy microstructures (C).

Table 2 summarizes the results from linear fits for five eutectic alloy systems, including T_onset_, intercepts, slopes, and 0.99 confidence boundaries. The recommended melting temperatures of RGTMs in Table 1-C match the assessed equilibrium eutectic temperatures in Table 2. The differences between the upper and lower bounds of 0.99 confidence intervals are from ± 0.24 °C of the Fe-Nb system to ± 0.86 °C of the Ru-Si system. A Comparison of the results in Table 1 and Table 2 indicates that the measurement error of RGTMs is within ± 1.00 °C.

**Table 2:** Summary of the linear fit for eutectic and off-eutectic alloys

Eutectic composition/mole fraction %	Eutectic reaction	Equilibrium eutecic temperature/°C	Intercept temperature/°C	Lower bound of intercept (confi. level: 0.99)/°C	Upper bound of intercept (confi. level: 0.99)/°C	Slope/min	Lower bound of slope (confi. level: 0.99)/min	Upper bound of slope (confi. level: 0.99)/min
Ni-41Nb	**L NbNi**_**3**_** + Nb**_**7**_**Ni**_**6**_	**1181**	**1.177E + 03**	1.176E + 03	1.177E + 03	** − 1.567E-01**	− 1.990E-01	− 1.140E-01
Co-13Nb	**L (Co) + Co**_**3**_**Nb**	**1240**	**1.237E + 03**	1.236E + 03	1.237E + 03	** − 1.444E-01**	− 1.920E-01	− 9.700E-02
Fe-10Nb	**L (Fe) + Fe**_**3**_**Nb**	**1372**	**1.368E + 03**	1.368E + 03	1.368E + 03	** − 7.790E-02**	− 9.900E-02	− 5.700E-02
Rh-23.5Si	**L (Rh) + Rh**_**2**_**Si**	**1421**	**1.417E + 03**	1.417E + 03	1.417E + 03	** − 1.510E-01**	− 1.990E-01	− 1.030E-01
Ru-28Si	**L (Ru) + Ru2Si**	**1535**	**1.531E + 03**	1.530E + 03	1.532E + 03	** − 1.860E-02**	− 1.160E-01	7.900E-02

The DSC heating curves (B) in Figs. 5–9 show acceptable linear slopes and quantifiable peak areas as a function of the heating rate. The peak slope and area correspond to the alloy melting reaction and the total thermal evolution of melting, respectively. The DSC curves show that as the heating rates increase, the peak areas increase, and the melting temperatures decrease. Most of all, the peaks maintain consistent slopes independent of the heating rates except for Ru-28Si. These properties of RGTMs show promise for further investigation into calibrating heat evolution in measurements.

The microstructures (C) in Figs. 5–9 also show a large fraction of the well-distributed lamellar eutectic structure and changes in primary phases depending on the alloy compositions. Adjustments of alloy compositions are needed to minimize the presence of primary phases. Unlike other materials, the eutectic microstructure of Ru-28Si is acicular, and the melting curve sometimes shows irregularities; however, the T_onset_ can be determined.

### Comparison of the calibration fits between the elemental standards and RGRMs

**Fig. 10:**
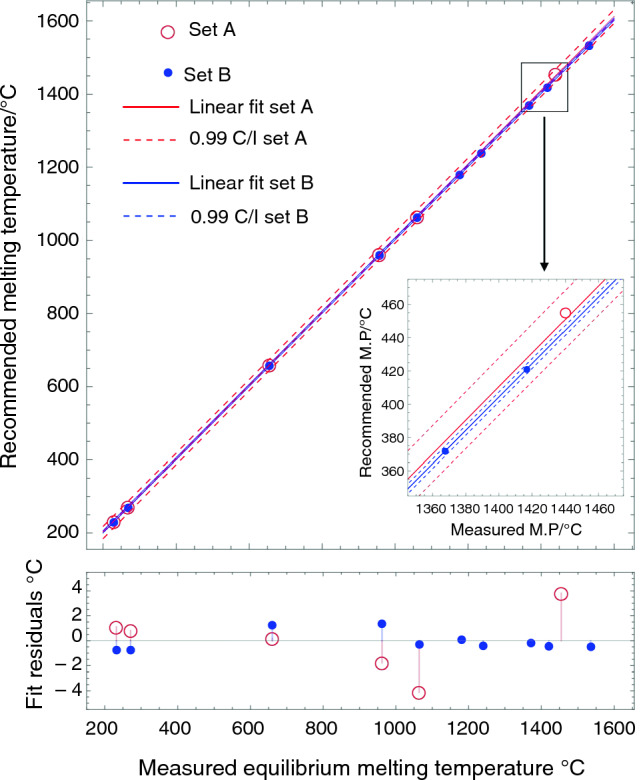
**A** Assessments of the equilibrium eutectic temperatures using Ru-27Si, Ru-28Si, Ru-29Si, and Ru-31Si alloys; **B** the DSC results of Ru-27Si, Ru-28Si, and Ru-29Si alloys depending on the heating rates; and **C** typical microstructures of Ru-27Si, Ru-28Si, and Ru-29Si alloys

Figure 10 shows the linear fit results for test specimen set A with red open circles and lines. This set includes Sn, Bi, Al, Ag, Au, and Ni. The set B results are shown as blue-filled circles and lines. Set B includes Sn, Bi, Ag, Au, Ni-41Nb, Co-13Nb, Fe-10Nb, Rh-23Si, and Ru-28Si. The solid lines represent the linear fits, while the dotted lines represent the 0.99 confidence boundaries. Both fit results for A and B exhibit a better R-squared value than 0.9999 and show minimal discrimination. However, there are differences in the fit residuals and the estimated variances, showing improvements in precision above 1000 °C. The estimated variances of set A and set B are 9.16 and 0.65 °C, respectively, indicating calibration accuracy improvement. Note that the extra oxygen trap insert was not utilized for these measurements to compare the results under normal test conditions.

## Discussion

The suggested method for determining T_onset_ is a simple and reliable interpolation method when the baseline of T versus ΔT is linear, as exhibited in the heating curves of elemental standards and RGTMs. However, when the baseline is curved due to the phase transition of alloys, this method is limited in accuracy, similar to determining T_extrap_ in Fig. 1. To address this limitation, the residuals were evaluated by extrapolating between the measured curves and a non-linear quadratic fit from the selected region before the melting. T_onset_ was then determined using the suggested strategy from the evaluated residuals. However, there were discrepancies in the T_onset_ depending on the selection of the region, requiring further development in the strategy to determine the T_onset_ in the case of the curved baseline of alloys.

When determining the melting temperatures of RGTMs, the elemental standards are employed to determine the calibration factor precisely and accurately. The oxygen-getter material was inserted when measuring Ni and Co standards. The calibration linear fit was validated using three other alloys, and then the melting temperatures of RGTMs were determined from the calibration linear fit. However, the melting temperature of Ru-31Si was estimated as 1535 ℃ by extrapolating the calibration linear fit. This extrapolation was utilized because the melting temperature of Ru-28Si is close to the Co melting temperature (1495 ℃), and the calibration linear fit shows good linearity. This extrapolated melting temperature is close to the published eutectic temperature (between 1536 ℃ and 1541 ℃) of Liquid ⬄ (Ru) + Ru_2_Si [13–16]. Therefore, the estimated melting temperature of Ru-28Si at 1535 ℃ is considered acceptable.

The terms “sensitivity” and “resolution” in DSC/DTA measurements generally refer to the instrument’s ability to detect energy changes and to distinguish between transitions that occur close in temperature, respectively. Increasing the sensitivity often results in a trade-off with the resolution; e.g., using a larger sample and a faster heating rate for a high sensitivity leads to a decrease in the resolution [17, 18]. Nevertheless, when calibrating temperature under given DSC/DTA conditions, it is recommended to use sample masses between 70 and 150 mg to improve sensitivity, which also allows for a unique mean value and less error (3σ ≲ 0.3) in the melting points. If the sample mass is smaller than 50 mg, the average melting point starts to increase with a larger distribution of errors (3σ ≳ 1.9).

In addition, the microstructure with finer interlamellar spacing and a minimal primary phase fraction is vital for an effective melting signal. To achieve this fine eutectic microstructure and a minimal amount of primary phase, a faster cooling rate is necessary. However, to achieve a fine eutectic microstructure without the primary phase, so-called true eutectic microstructure, adjusting the alloy composition to be close to the *true eutectic microstructure composition* is essential. For instance, in Fig. 7C, there are notable changes in the primary phases from (Co; FCC) to (Co_3_Nb; C36), depending on the alloy composition. If the presence of the primary phases is considered, the *true eutectic microstructure composition* should form between Co-13Nb and Co-15Nb.

## Conclusions

Five RGTMs, Ni-41Nb (1181 °C), Co-13Nb (1240 °C), Fe-10Nb (1372 °C), Rh-23.5Si (1421 °C), and Ru-31Si (1535 °C), have been developed and validated to cover the temperature range between 1100 and 1600 °C. This set of RGTMs improves the estimated variance for temperature measurements in this range from 9.16 to 0.65 °C. The melting temperature has been determined using a method that evaluates the equilibrium melting temperature based on heating rates. A new reliable method for determining T_onset_ has been developed to eliminate inaccurate human subjectivity and uncertain extrapolation methods.

## Footnotes

1 Certain commercial products or company names are identified here to describe our study adequately. Such identification is not intended to imply recommendation or endorsement by the National Institute of Standards and Technology, nor is it intended to imply that the products or names identified are necessarily the best available for the purpose.
